# Management of severe rhinophyma with electrocautery dermabrasion – A case report

**DOI:** 10.1016/j.ijscr.2020.06.039

**Published:** 2020-06-18

**Authors:** Brinda Chellappan, Jose Castro

**Affiliations:** Department of Surgery, Texas Tech University Health Sciences Center, El Paso, TX, United States

**Keywords:** Rhinophyma, Electrocautery, Dermabrasion, Case report, Rosacea

## Abstract

**Introduction:**

Rhinophyma is benign hypertrophic thickening of the skin and edema of the nasal pyramid. The affected tissue enlarges slowly before reaching its permanent size. The lobulated skin surface with hundreds of pores can become cosmetically embarrassing and cause significant psychosocial stress, anxiety, and depression for patients. In addition, extensive alar thickening can obstruct the external nasal valves making treatment necessary to alleviate respiratory issues. No consensus has been reached regarding management of rhinophyma and many surgeons follow the “to each his own technique” mindset. Our objective was to present a case report to support the use of electrocautery and dermabrasion as the mainstay of treatment.

**Presentation of case:**

Here we describe the case of a 62-year-old Caucasian male with a long-standing history of acne rosacea who developed severe rhinophyma overtime which lead to nasal obstruction and major cosmetic deformity. Electrocautery and dermabrasion in the operating room were utilized to obtain an outstanding cosmetic result and respiratory function improvement. Loop and Colorado cautery tips were used with cutting current to remove the hypertrophic skin and create a smooth contour. The patient tolerated the procedure well without any complications. The patient’s skin was scab-free with normal pigmentation by four weeks post-op. He was satisfied with the cosmetic outcome and reported substantial improvement in his breathing.

**Discussion:**

There have been several case reports published which describe using different surgical methods to treat rhinophyma including lasers, electrocautery dermabrasion, surgical blade, cryosurgery, and radio excision. The main limitations of laser therapy are imprecise tissue removal, risk of scarring, dyspigmentation, and bleeding. Other therapies such as surgical excision and skin grafts may require multiple procedures before obtaining a satisfactory cosmetic outcome.

**Conclusion:**

This case report supports electrocautery dermabrasion as the mainstay of treatment as it is a management technique which allows for smooth contouring, efficient hemostasis, more control in the operating room, and does not require multiple procedures.

## 1 Introduction

Rhinophyma is benign hypertrophic thickening with edema of the nasal pyramid skin. It is defined as the fourth stage of acne rosacea, an inflammatory dermatologic condition that often begins between the ages of 20 and 30 as facial flushing which is exacerbated by vasoactive substances such as caffeine and spicy foods [1,2]. Stage I of acne rosacea is also known as pre-rosacea with frequent blushing. Progression of the disease leads to Stage II which is described as permanent facial redness and telangiectasias. Stage III is deeper facial erythema with papules and pustules. The most advanced progression of the disease is stage IV which is characterized by phymatous changes (tissue hyperplasia) and/or ocular inflammation [3]. Phymatous changes most commonly occur on the nose (rhinophyma) but can also appear on the chin (gnatophyma), forehead (metophyma), ears (otophyma), and eyelids (blepharophyma). Treatment should begin at the earliest manifestations of the disease to prevent progression towards other stages and irreversible fibrotic changes [3,4].

Rosacea has a prevalence of at least 10% in Caucasian adults [5]. Women have acne rosacea 2–3 times more than men, however rhinophyma occurs almost exclusively in men greater than 40 years old [6,7]. Phymatous tissue usually enlarges slowly over a period of 10–15 years before reaching its permanent size. The lobulated skin surface has hundreds of pores which can become cosmetically embarrassing and cause significant psychosocial stress, anxiety, and depression for patients [8,9]. In addition, extensive alar thickening can obstruct the external nasal valves making treatment necessary to alleviate respiratory issues [2].

Topical therapies and oral medications such as metronidazole, isotretinoin, and tetracyclines work well for stage I or stage II rosacea by reversing skin vasodilation. Whereas, phymatous rosacea requires more intricate treatment via laser, electrocautery dermabrasion, surgical blade, cryosurgery, or radio excision [[9], [10], [11]]. Here, we describe a case of rhinophyma, in line with SCARE (Surgical CAse REport) 2018 criteria, that was treated successfully by electrocautery dermabrasion [12]. The surgery was performed by a board-certified plastic surgeon in an academic medical center. After the procedure, the patient reported satisfaction with the outstanding cosmetic result and respiratory improvement.

### 1.1 Histopathology

On histology, rhinophyma appears as hypertrophic sebaceous glands and connective tissue with signs of chronic inflammation [7,11]. It is beneficial to obtain a sample for histopathological evaluation because there may be underlying squamous cell carcinoma, basal cell carcinoma, or other angiomatous tumor that mimics rhinophyma [11]. These cases would require further follow-up and excision with appropriate margins. Although the malignant change to squamous cell carcinoma is rare, it is most likely due to chronic inflammation which is also seen in ulcers and burn scars. The telangiectasias of basal cell carcinoma may also be masked in rhinophyma, but one study concluded that basal cell carcinoma is more likely to arise from the normal surrounding skin rather than the phymatous skin [13,14].

### 1.2 Management

Surgical management in severe cases is the first line treatment for rhinophyma; however, it may be contraindicated in patients with cardiac failure, impaired hemostasis, or allergies to anesthesia [15]. In cases of surgical contraindications, laser and light treatments work well for general erythema and telangiectasias of rhinophyma. These techniques can easily be performed in an office setting. Common laser therapies include pulsed dye laser (PDL), copper vapor laser, CO_2_ laser, and intense pulsed light (IPL) to reach deeper vessels. PDL targets telangiectasias by affecting substance P, a neuropeptide which is responsible for the vascular changes seen in rosacea. The CO_2_ laser and erbium: yttrium-aluminum-garnet laser are considered to be first line lasers for rhinophyma management as they can carefully cut and vaporize skin while obliterating sebaceous glands and expression of sebum [2]. Laser therapies are successful methods of treatment since eradicating telangiectasias leads to decreased blood supply to the hypertrophic tissue and subsequent tissue reduction [15].

Rhinophyma is a gradual condition that presents at various levels of severity. Larger and more advanced cases may require several surgical procedures for treatment, whereas some minor cases can be treated simply with a sterile single-blade disposable razor [16]. The technique of combined surgical excision and electrocautery allows for debulking of excess tissue layer by layer, precise hemostasis, and contouring of an aesthetic nasal shape [9,11]. Dermabrasion, radiofrequency and loop cautery especially have been shown to be effective in creating a smooth, contoured appearance [2,17,18]. Regardless of which laser or cautery option is chosen, care must be taken to leave the lower part of the pilosebaceous unit intact to allow for adequate healing via secondary intention [16]. Another treatment option is a two-step reconstruction by using acellular dermal matrix and a full thickness skin graft to replace the lower half of the nose [19].

In our case, we describe use of electrocautery dermabrasion to successfully remove the excess phymatous skin, improve respiration, and create an aesthetic contour of the nose all in a single procedure.

## 2 Case report

Our patient was a 62-year-old Caucasian male with a long-standing history of acne rosacea who developed severe rhinophyma over several years which lead to nasal obstruction and major cosmetic deformity. He did not seek previous treatment as the skin changes were so gradual that it was not noticed until the rhinophyma reached significant size. Many patients in fact do not notice the skin changes until they see an old picture of themselves. In pre-op evaluation, his affected skin was noted to be porous, edematous, and had a rough texture.

**Fig. 1:**
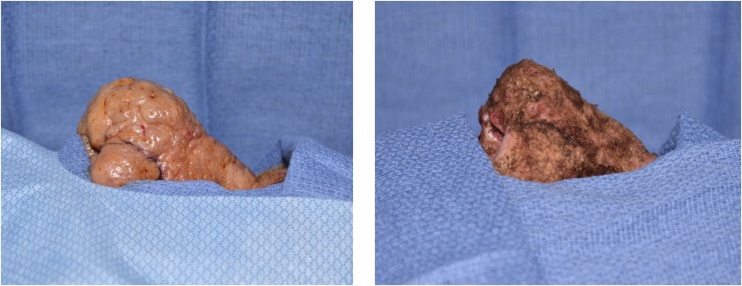
Left: Rhinophyma before electrocautery dermabrasion. Right: Immediately after electrocautery dermabrasion and prior to application of bacitracin ointment in the operating room.

The decision was made to take the patient to the operating room (OR) given the severity of the disease and the possibility of significant blood loss at the time of excision. The patient was placed in a supine position for induction of general endotracheal anesthesia. Preoperative Cefazolin was given IV for infection prophylaxis. In the OR, a loop cautery tip (Bovie ¾ short shaft thin wire disposable electrode, Bovie Medical Antioch, TN) was utilized to perform meticulous dissection layer by layer of the hypertrophic skin of the radix down to the tip, lateral nasal walls, and nasal alae. This was done with multiple passes using cutting current to create a smooth contour. Multiple cysts were encountered during removal of excess skin at the dorsal tip and nasal alae and they were drained simultaneously. Colorado cautery tip (Stryker, Kalamazoo, MI) was then used to level all irregularities of the skin from the excision and to control hemostasis. A field block with 0.5% Marcaine plain was placed for postoperative pain control. Estimated blood loss was 20 cc’s. The total procedure length was less than 90 min. Topical bacitracin ointment was applied generously to the treated area. The patient was admitted to the hospital for 23-h observation for adequate postoperative pain management, wound care education and to monitor blood loss of the raw de-epithelized areas (Fig. 1).

The patient had his first follow-up appointment in the clinic two weeks after discharge from the hospital. He described substantial improvement in breathing and satisfaction with the cosmetic result. The patient reported that he used to feel embarrassed about the way his nose appeared that even when stopped at a traffic light, he would cover his nose with his hand to avoid the stares from nearby cars. Now he has a new sense of self-confidence and no longer feels the need to hide his nose.

**Fig. 2:**
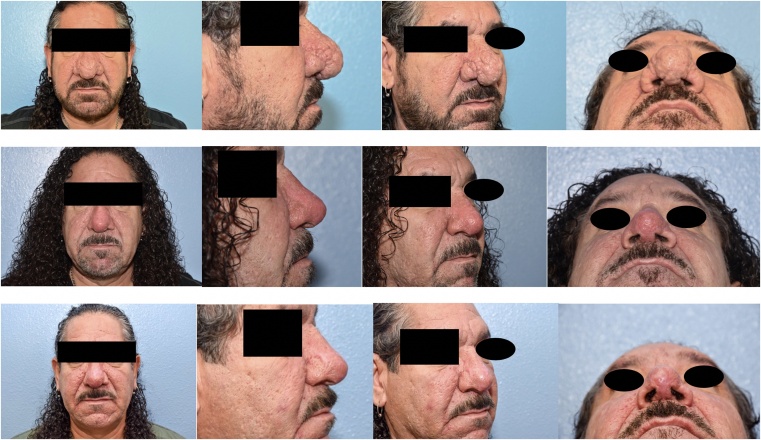
Top row: Pre-operative consultation. Middle Row: One month post-procedure with significant improvement in breathing. Bottom row: Two months post-procedure with normal skin pigmentation.

There was some skin scabbing as expected, however by four weeks post-op, the patient’s skin was scab free with normal pigmentation. The patient was advised to avoid direct exposure to sunlight, and the importance of SPF 30 sunscreen use was emphasized to prevent skin hyperpigmentation (Fig. 2).

## 3 Discussion

The pathophysiology of rosacea remains unclear, but certainly involves vascular and inflammatory factors. Tissue damage occurs as a result of vasoactive substances such as serotonin, prostaglandins, and substance P [4]. Induction of local transforming growth factor (TGFβ1) also leads to fibrosis and thickening of the skin. The increased edema predisposes the skin to be colonized by a parasite called *Demodex Folliculorum,* which may contribute to the etiology of the disease. These mites are about 0.4 mm in size and live in pores and hair follicles [2,4,7]. Considering vascular and inflammatory processes, differential diagnoses for rosacea and rhinophyma include contact dermatitis, sarcoidosis, cutaneous lupus, carcinoid syndrome, acne vulgaris, squamous cell carcinoma, and basal cell carcinoma [5,13,14].

Rhinophyma is mistakenly associated with alcohol abuse, thus worsening the social stigma [7,16]. Often, the presence of dermatologic conditions in film are depicted in villainous characters, which may also contribute to the prejudices and stigma applied by the general public towards patients [20]. In conclusion, rhinophyma does not affect survival, but still has a major cosmetic and psychosocial impact, with severe cases even leading to respiratory difficulty. Thus, significant rhinophyma is a disease best managed in the field of plastic and reconstructive surgery.

There have been several case reports published using different surgical methods to treat rhinophyma. Nonetheless, no consensus has been reached and many surgeons follow the “to each his own technique” mindset [7]. Our case strongly advocates for loop electrocautery dermabrasion to be the mainstay of treatment as it allows for the best control of hemostasis in the operating room and final cosmetic result. Electrocautery dermabrasion is also less invasive compared to surgical excision and only requires one trip to the operating room.

The main limitations of laser therapy are imprecise tissue removal, risk of scarring, dyspigmentation, and bleeding which can obstruct visualization [2,9]. Compared to electrocautery, CO_2_ lasers do not allow for as precise ablation of phymatous tissue. One study described use of a “gopher sign” to determine adequate depth of CO_2_ laser ablation since once glandular content is expressed, the remaining tissue resembles a gopher reaching out of its burrow. However, this is a clinical sign that may require extra caution especially with limited clinical experience to prevent over ablation, hypertrophic scarring, and delayed wound healing [21]. Hemostasis is better achieved with bipolar electrocautery compared to laser treatment. Furthermore, meticulous hemostasis is necessary to prevent hematoma and post-operative bleeding [7,11].

Two-step reconstruction by using acellular dermal matrix and full thickness skin graft is another option for large rhinophymas, however the resorption rate of the matrix and the retraction rate of the skin graft are unpredictable. This could lead to a “step-like effect” due to the difference in thickness of the reconstructed tissue compared to the surrounding skin. One case report that described this technique required additional surgery for rhinoplasty with satisfactory outcome [19].

Emerging therapies have become more prominent like botulinum toxin and there is current *in vitro* research regarding use of Tamoxifen (an anti-estrogen medication) as potential treatment. Tamoxifen can inhibit fibrosis seen in Dupuytren contracture and may help prevent early stage rosacea from progressing to rhinophyma by acting on fibroblasts and decreasing the production of TGFβ2 (a transforming growth factor) [2].

## 4 Conclusion

As of now, surgical excision and/or electrocautery with dermabrasion seem to be the most successful treatments for sizeable rhinophymas. We support electrocautery dermabrasion as the mainstay of treatment as it is a management technique which allows for smooth contouring, efficient hemostasis, more control in the operating room, and does not require multiple procedures.

## Declaration of Competing Interest

None declared. The authors have no financial, consultative, institutional and other relationships that might lead to bias or conflict of interest.

## Sources of funding

None declared.

## Ethical approval

None declared.

## Registration of research studies

NA.

## Author contribution

Brinda Chellappan - literature review and writing of manuscript.

Jose Castro Garcia - revision and approval of final manuscript.

## Guarantor

Jose Castro Garcia, MD.

## Provenance and peer review

Not commissioned, externally peer-reviewed.
